# Interferon-stimulated circHOMER1 attenuates antiviral innate immunity

**DOI:** 10.1128/mbio.01497-25

**Published:** 2025-07-15

**Authors:** Ao Zhang, Ranran Yao, Shijin Geng, Haiying Wang, Rong-Chun Tang, Hengxiang Yu, Rui Xu, Hong-Yan Chen, Yunxuan Zhou, Lan Zhang, Jun Zhang

**Affiliations:** 1 Department of Immunology, School of Basic Medical Sciences, NHC Key Laboratory of Medical Immunology, Medicine Innovation Center for Fundamental Research on Major Immunology-related Diseases, Peking University 601349, Beijing, China; 2 Department of Biochemistry and Molecular Biology, School of Basic Medical Sciences, Peking University 208326 , Beijing, China; Washington University in St. Louis, St. Louis, Missouri, USA

**Keywords:** circHOMER1, ceRNA, miR-145-3p, innate immunity, RIG-I

## Abstract

In an initial screening of virus-induced differentially expressed circRNAs, circHOMER1 was significantly upregulated. However, its role in antiviral innate immunity remained unclear. circHOMER1 was upregulated in response to a range of stimuli, including RNA viruses, DNA viruses, as well as type I IFNs. Overexpression of circHOMER1 reduced the virus-induced type I IFN signaling pathway and promoted viral replication. Consistently, knockdown of circHOMER1 yielded opposite consequence. Mechanistically, circHOMER1 acted as a competitive endogenous RNA (ceRNA) for miR-145-3p, controlling the mRNA level of OTU domain-containing protein 7B (OTUD7B), thereby downregulating interferon regulatory factor 3 (IRF3) expression. Furthermore, circHOMER1 was found to disrupt the interaction between Ras GTPase-activating protein-binding protein 1 (G3BP1) and retinoic-acid-inducible protein I (RIG-I), impairing the detection of viral RNAs by RIG-I. Notably, the concordant immunosuppressive effects of circHOMER1 were observed in mouse infection models. These findings identify circHOMER1 as a novel interferon-stimulated circRNA that serves as a negative modulator of the antiviral innate immune response and is essential for immune homeostasis.

**IMPORTANCE:**

A growing body of evidence suggests that circRNAs are involved in various physiological or pathological processes. Through circRNA microarray screening of virus-induced differentially expressed circRNAs, we identified an upregulated circRNA, circHOMER1. circHOMER1 is abundantly expressed in the brain. The known functions of circHOMER1 have been focused on the nervous systems and the related diseases. Its functions in the immune system are not discovered. In this study, we found that circHOMER1 is upregulated by viral infection, interferon treatment, etc. circHOMER1 can negatively modulate virus-induced type I IFN signaling by acting as a ceRNA or other mechanism. The regulatory function of circHOMER1 in innate immunity is critical for immune homeostasis. The dysregulation of circHOMER1 may be associated with virus encephalitis or other related diseases. circHOMER1 may be a potential therapeutic target of virus-induced infections and autoimmune diseases.

## INTRODUCTION

Circular RNAs (circRNAs), a class of non-coding RNAs, are generated through back-splicing of pre-mRNA transcripts, forming covalently closed loop structures without canonical 5′ caps and 3′ polyadenylated tails (1). circRNAs are ubiquitously and abundantly expressed in mammalian cells, some of which with cell or tissue-specific expression (2). circRNAs are characterized by enhanced stability and resistance to exonuclease digestion (2). Recently, circRNAs have been shown to be translatable, with a subset capable of utilizing internal ribosome entry site (IRES) or m^6^A modification for protein synthesis (3). Accounting for over 10% of the expressed transcripts in the human genome, circRNAs have been reported to be involved in physiological or pathological processes including the antiviral innate immune response (4, 5).

In uninfected cells, the nuclear factor 90 and 110 (NF90/NF110) complex critically regulates the biogenesis of nascent circRNAs. Endogenous circRNAs bind the double-stranded RNA-dependent protein kinase (PKR), a pattern recognition receptor (PRR) for double-stranded RNA (dsRNA), potentially mitigating aberrant PKR-mediated immune recognition in quiescent states (6). Viral infection induces biphasic regulation of circRNAs, marked by suppressed synthesis and enhanced degradation. Exposure to vesicular stomatitis virus (VSV) or poly(I:C) triggers cytoplasmic translocation of nuclear NF90/NF110, thereby inhibiting circRNA production. Concurrently, RNase L-mediated circRNA degradation and PKR release for foreign nucleic acid detection are activated (6). It is noteworthy that certain circRNAs can also be upregulated after infection. For example, Kaposi’s sarcoma-associated herpesvirus (KSHV) infection promotes an increase in 98 or 254 circRNAs in HUVECs or MC116 cells, respectively (7); whereas 414 circRNAs are upregulated in SARS-CoV-2-infected PBMCs (8), and 159 circRNAs are increased in influenza A virus (IAV)-induced lung injury (9). Furthermore, virally encoded circRNAs may enhance viral propagation and antagonize the antiviral innate immunity (10).

Differentially expressed circRNAs modulate antiviral innate immunity through diverse mechanisms (4, 5). These include functioning as miRNA sponges to regulate host gene expression, interacting with RNA-binding proteins (RBPs) to fine tune their activity, and encoding functional peptides with antiviral or pro-viral properties. Certain circRNAs govern apoptosis to restrict viral replication (11). Under viral stress, circRNAs further integrate into stress granules to coordinate cellular responses (12). Although the role of circRNAs in innate immunity regulation is well-documented, the functional characterization of infection-induced circRNAs remains limited in comparison to the large number of circRNAs differentially expressed during viral infection, implying the possibility of unidentified circRNAs that modulate antiviral innate immunity.

circHOMER1 (hsa_circ_0006916), a brain-enriched circRNA, is formed by back-splicing of exons 2-5 of *pre-HOMER1B* (13). Lacking an internal ribosome entry site (IRES) for translation capability, its biogenesis is facilitated by the RNA-binding proteins FUS (fused in sarcoma) and eIF4A3 (eukaryotic translation initiation factor 4A3), with upstream regulation by cAMP-response element-binding protein (CREB) (13, 14). Current functional studies of circHOMER1 are focused on the nervous system and related diseases (13, 15–19). circHOMER1 is downregulated in female patients with Alzheimer’s disease (16). It is required for synaptic plasticity and neuronal activity (16). Treatment with methamphetamine (METH) increases circHOMER1 levels (20). Inhibition of circHOMER1 reduces METH-induced neurotoxicity (21). In addition, circHOMER1 is upregulated in cancers such as hepatocellular carcinoma and colorectal cancer and has pro-tumor potential. Lidocaine reduces its expression in colorectal cancer cells (22, 23). Nevertheless, its immunoregulatory functions remain unexplored.

A circRNA microarray screening revealed that circHOMER1 was upregulated by Sendai virus (SeV) infection, suggesting a potential role for circHOMER1 in antiviral innate immunity. Further studies suggested that a variety of triggers, such as RNA viruses, DNA viruses, and type I IFNs, can upregulate circHOMER1. Overexpression or knockdown of circHOMER1 indicated that circHOMER1 was a novel negative modulator in antiviral innate immune response. It can act as a sponge for miR-145-3p, stabilizing OTUD7B mRNAs and driving p62-dependent degradation of IRF3. Concurrently, circHOMER1 restricted the binding of G3BP1 to RIG-I, impairing the ability of RIG-I to sense viral RNAs, ultimately dampening antiviral innate immunity.

## RESULTS

### circHOMER1 is a differentially expressed circRNA upon viral infection

**Fig 1:**
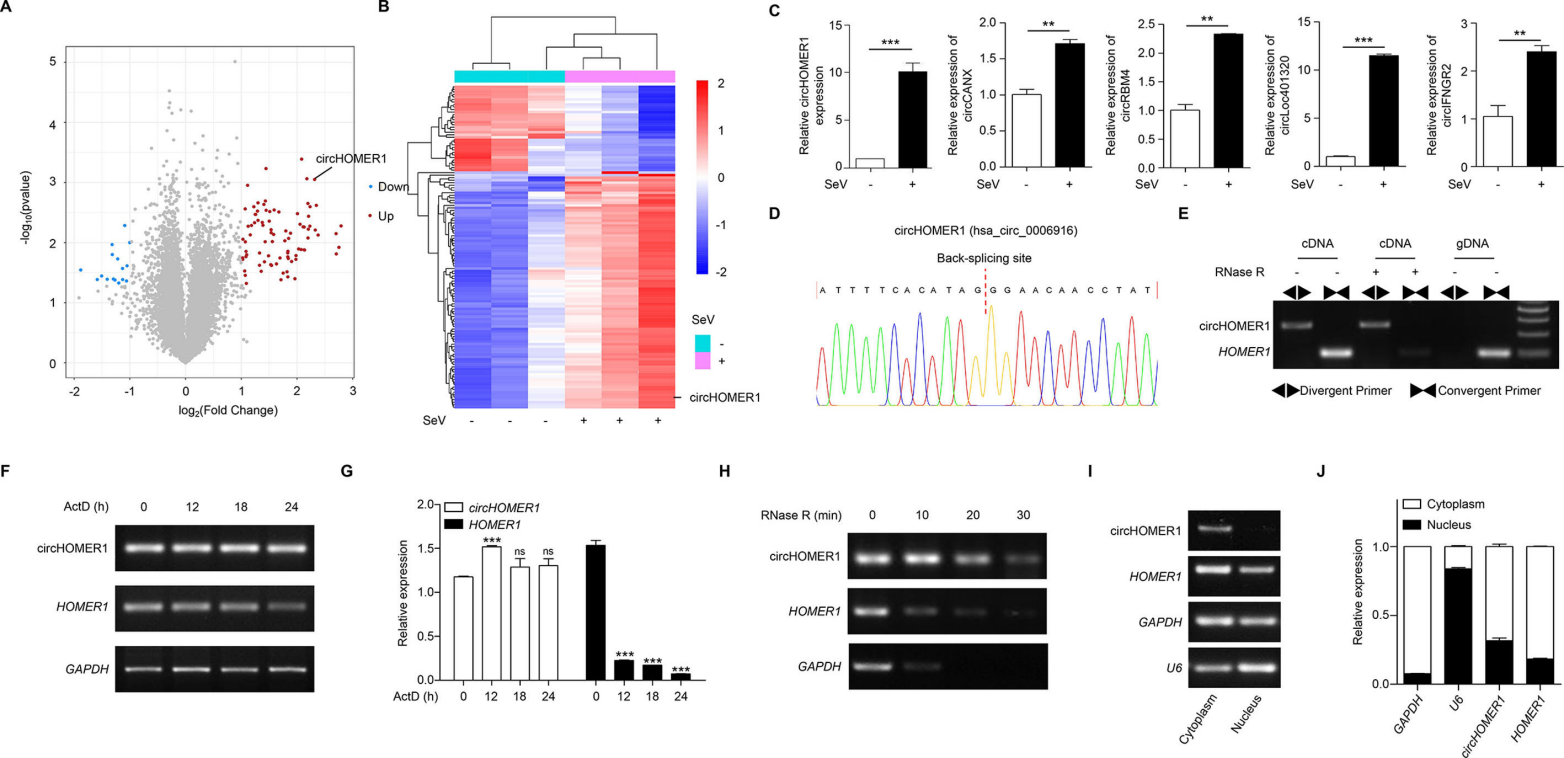
circHOMER1 is a differentially expressed circRNA upon viral infection. (**A and B**) Volcano plots (A) and heatmap (B) showing the 82 most upregulated circRNAs and 20 most downregulated circRNAs with the thresholds of *P*-value < 0.05 and multiplicity of difference > 2; (**C**) qRT-PCR was performed to analyze the relative expression of circHOMER1, circCANX, circRBM4, circLoc401320, and circIFNGR2 in HEK293 cells at 12 h after SeV infection; (**D**) the back-splicing site of circHOMER1 identified by sanger sequencing; (**E**) end-point PCR was conducted to analyze circHOMER1 and its linear transcript in cDNA and gDNA amplified by convergent and divergent primers; (**F and G**) end-point PCR and qRT-PCR analyses of the relative amount of circHOMER1 and its linear transcript in HEK293T cells treated by actinomycin D (3 µg/mL); (**H**) end-point PCR analyses were conducted to identify the stability of circHOMER1 and its linear transcript in HEK293T cells after RNase R treatment; (**I and J**) end-point PCR and qRT-PCR were conducted to analyze circHOMER1 amount in the cytoplasmic and nuclear fractions of HEK293T cells. U6 and GAPDH were used as positive controls in the nucleus and cytoplasm, respectively.

To obtain a landscape of differentially expressed circRNAs upon RNA virus infection, we performed circRNA microarray profiling in human embryonic kidney cell line 293 (HEK293) cells infected with SeV. Using the human CircRNA Array V2 (8 × 15K, Arraystar) with a total of 10,926 circRNA probes, we identified 82 upregulated and 20 downregulated circRNAs with a threshold of *P*-value < 0.05 and a fold change cutoff of 2 by volcano plots (Fig. 1A) and heatmap (Fig. 1B). Validation by qRT-PCR confirmed induction of five candidate circRNAs after SeV infection (Fig. 1C), with circHOMER1 showing marked upregulation after SeV infection (Fig. 1C), which was selected for further study.

circHOMER1, a 522-base circular RNA, is derived from the back-splicing of exons 2–5 of the *HOMER1* gene on human chromosome 5 at positions 78734832 to 78752841. The back-splicing site was verified by Sanger sequencing (Fig. 1D). circHOMER1 PCR products can only be amplified with divergent primers using cDNA as the template (Fig. 1E). The stability of circHOMER1 was also examined after treatment with actinomycin D (ActD). It remained largely unaltered up to 24 h after treatment (Fig. 1F and G). Due to the lack of 5′ and 3′ ends, circRNAs are resistant to RNase R digestion (2). Upon treatment with RNase R, the expression of circHOMER1 remained relatively stable, whereas the expression of linear *HOMER1* was reduced (Fig. 1H). Subcellular fractionation revealed that circHOMER1 was present in both the nucleus and the cytoplasm, with a predominant cytoplasmic localization (Fig. 1I and J).

### circHOMER1 is upregulated by diverse stimuli

**Fig 2:**
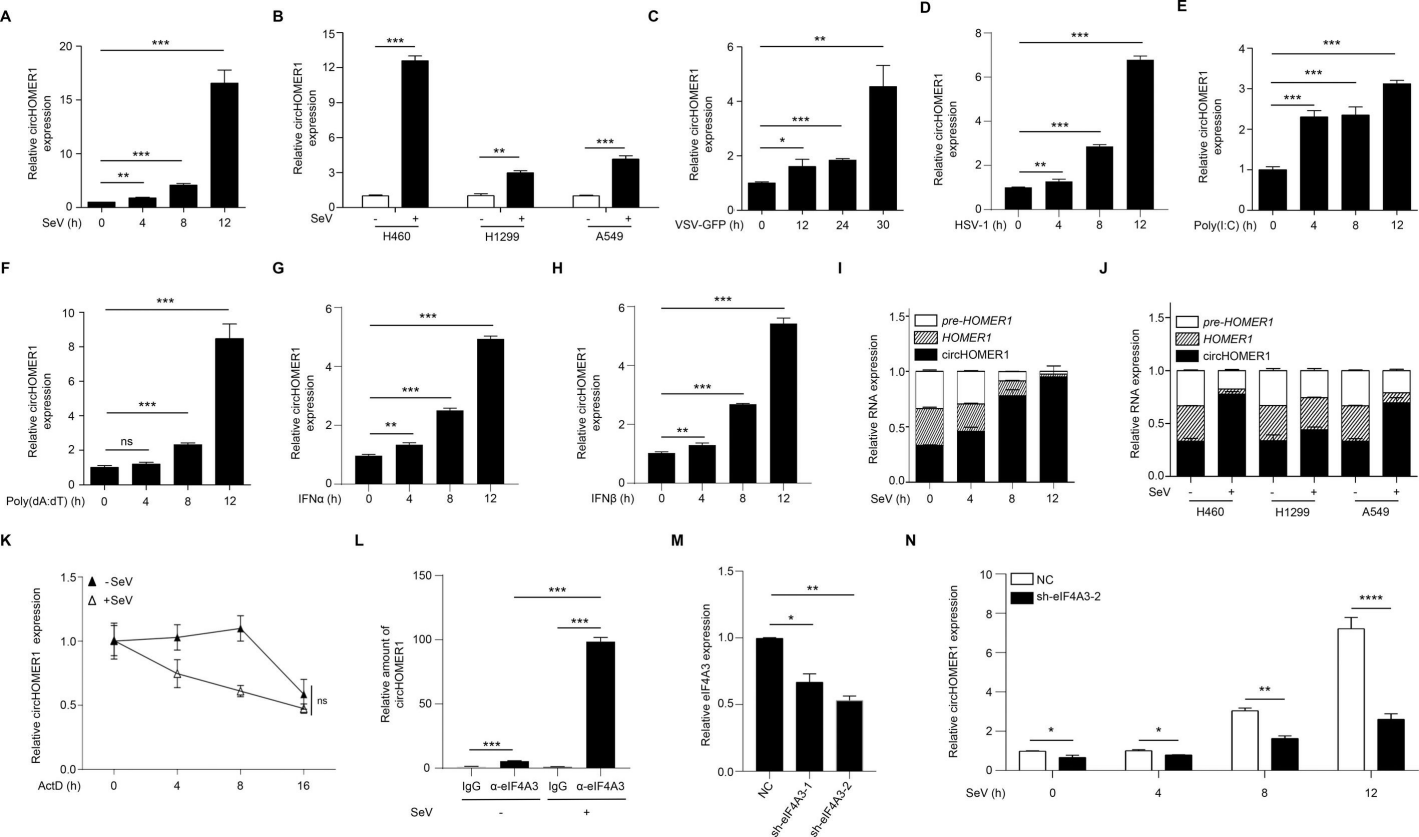
circHOMER1 is upregulated by diverse stimuli. (**A**) The relative expression of circHOMER1 was analyzed by qRT-PCR in HEK293T cells after SeV infection for indicated hours; (**B**) qRT-PCR was performed to analyze the relative expression of circHOMER1 in H460, H1299, and A549 cells at 12 h after SeV infection; (**C**) the relative expression of circHOMER1 was analyzed by qRT-PCR in HEK293T cells after VSV-GFP infection for indicated hours; (**D**) qRT-PCR was performed to analyze the relative expression of circHOMER1 in HeLa cells after HSV-1 infection for indicated hours; (**E and F**) the relative expression of circHOMER1 was analyzed by qRT-PCR in HEK293T cells transfected with poly(I:C) and poly(dA:dT) for indicated hours; (**G and H**) qRT-PCR was performed to analyze the relative expression of circHOMER1 in HEK293T cells stimulated by IFNα (**G**) (10 ng/mL) and IFNβ (**H**) (10 ng/mL) for indicated hours; (**I**) the relative expression of *pre-HOMER1*, *HOMER1,* and circHOMER1 was analyzed by qRT-PCR in HEK293T cells after SeV infection for indicated hours; (**J**) the relative expression of *pre-HOMER1*, *HOMER1,* and circHOMER1 was analyzed by qRT-PCR in H460, H1299, and A549 cells at 12 h after SeV infection; (**K**) qRT-PCR analyses of the relative expression of circHOMER1 in HEK293T cells treated by actinomycin D (3 µg/mL) followed by infection with or without SeV infection for indicated hours. (**L**) RIP was performed to analyze the interaction of circHOMER1 and eIF4A3 in HEK293T cells; (**M**) the knockdown effect of eIF4A3-shRNA was examined by qRT-PCR analyses of the relative expression of circHOMER1; (**N**) qRT-PCR analyses of the relative expression of circHOMER1 in HEK293T cells transfected with NC or sh-eIF4A3-2, which subsequently infected with SeV for indicated hours.

The above data showed that circHOMER1 was markedly increased in HEK293 cells upon SeV infection (Fig. 1C). We then sought to determine whether circHOMER1 was also upregulated in response to different stimuli or in other cells. Upon SeV infection, circHOMER1 was also upregulated in HEK293T cells (Fig. 2A). circHOMER1 has previously been shown to be expressed in lung cancer epithelial cell lines (24). After SeV infection, circHOMER1 was upregulated in lung H460, H1299, and A549 cells, with the highest upregulation observed in H460 cells (Fig. 2B). In addition, circHOMER1 was upregulated in HEK293T cells stimulated with vesicular stomatitis virus-green fluorescent protein (VSV-GFP) (Fig. 2C) and in human cervical carcinoma cell line HeLa cells stimulated with herpes simplex virus type 1 (HSV-1) (Fig. 2D), as well as by treatment with RNA virus mimics poly(I:C) (Fig. 2E), DNA virus mimics poly(dA:dT) (Fig. 2F), and type I IFNs (Fig. 2G and H). circRNAs often compete with the linear transcripts for the shared pre-mRNAs (25). Based on the reported cross-regulation between circHOMER1 and linear *HOMER1* (13), we investigated their biogenetic interplay after viral stimulation. It was shown that after SeV infection, there was an increase in the biosynthesis of circHOMER1, while there was a decrease in the linear transcript *HOMER1* and the pre-mRNA in both HEK293T and lung cancer epithelial cell lines (Fig. 2I and J).

The half-life of circHOMER1 was determined upon viral stimulation. Upon SeV infection, the half-life of endogenous circHOMER1 was not shortened compared to the uninfected group in HEK293T cells (Fig. 2K), indicating that viral infection did not lead to the degradation of circHOMER1. Given the established role of RBP eIF4A3 in neuronal circHOMER1 biogenesis (13), we hypothesized its involvement in the increased expression of circHOMER1 after viral infection. SeV infection was shown to strengthen the interaction between eIF4A3 and circHOMER1 (Fig. 2L). Whereas, eIF4A3 knockdown abolished SeV infection-induced circHOMER1 biogenesis (Fig. 2M and N). Taken together, circHOMER1 is a responsive circRNA to multiple stimuli, including viral infection and type I IFNs.

### circHomer1 is upregulated by viral infection

**Fig 3:**
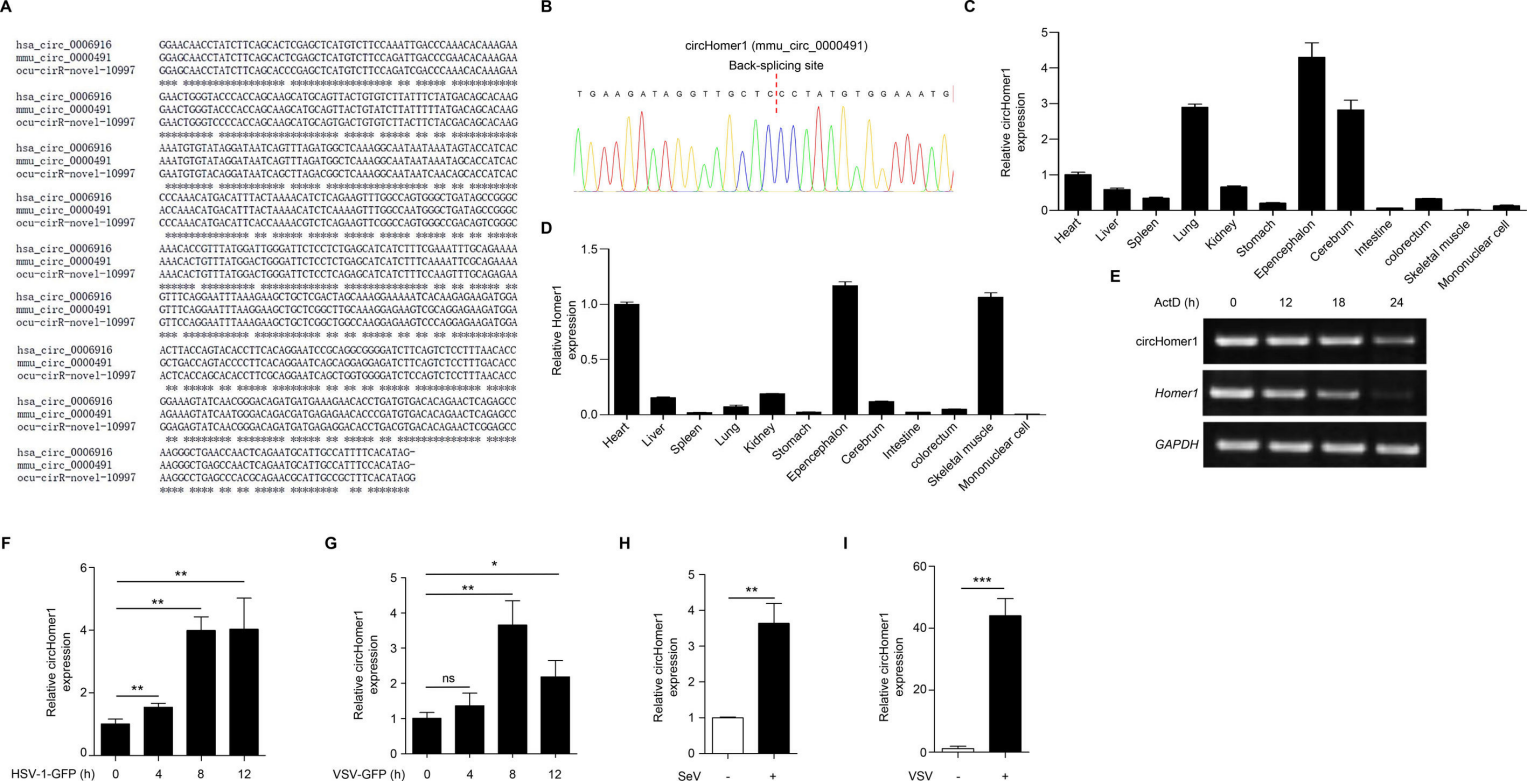
circHomer1 is upregulated by viral infection. (**A**) Sequence alignment of circHomer1 in human, mouse, and rabbit through circFunBase database analyses; (**B**) the back-splicing site of circHomer1 verified by sanger sequencing; (**C and D**) qRT-PCR analyses of the relative expression of circHomer1 (C) and *Homer1* (D) in organs of mouse and mononuclear cells; (**E**) end-point PCR analyses of the relative amount of circHomer1 and its linear transcript in mouse iBMDM cells treated by actinomycin D (3 µg/mL); (**F–H**) qRT-PCR analyses of the relative amount of circHomer1 in mouse iBMDM cells (F and G) or peritoneal macrophages (H), after DNA or RNA viral stimulation in indicated hours, β-actin was used as a basal control. (I) qRT-PCR analyses of the relaive amount of circHomer1 in the brain tissues of viral encephalitis model. β-Actin was used as a basal control.

To assess cross-species conservation, CircFunBase analysis revealed *HOMER1* gene-derived circRNA in human (hsa_circ_0006916), mouse (mmu_circ_0000491, hereafter *circHomer1*), and rabbit (ocu-cirR-novel-10997), with human circHOMER1 sharing 93% and 89% sequence identity to mouse and rabbit orthologs, respectively (Fig. 3A). Sanger sequencing confirmed the sequences flanking the circularization splicing sites of mouse circHomer1 (Fig. 3B), which was nearly identical to that of circHOMER1, according to the sequence alignment in Fig. 3A.

circHomer1 showed a tissue-specific enrichment that differed from its linear counterpart. circHomer1 was predominantly expressed in the lung and brain tissues (Fig. 3C). In contrast, the linear transcript was highly expressed in epencephalon, heart, and skeletal muscle (Fig. 3D). Mirroring human circHOMER1, mouse circHomer1 showed increased stability in ActD-treated iBMDM compared to the mouse *Homer1* counterpart (Fig. 3E). Infection with DNA virus herpes simplex virus type 1-green fluorescent protein (HSV-1-GFP) or RNA virus VSV-GFP and SeV induced upregulation of circHomer1 in both immortalized bone marrow derived macrophages (iBMDM) (Fig. 3F and G) and primary peritoneal macrophages (Fig. 3H), which are important effector innate immune cells for viral clearance. Moreover, a VSV-induced encephalitis model was established and showed a significant increase in circHomer1 expression in the brain (Fig. 3I). These findings demonstrate that circHOMER1 is an evolutionarily conserved, viral upregulated circRNA across mammals.

### circHOMER1 inhibits virus-induced innate immunity

**Fig 4:**
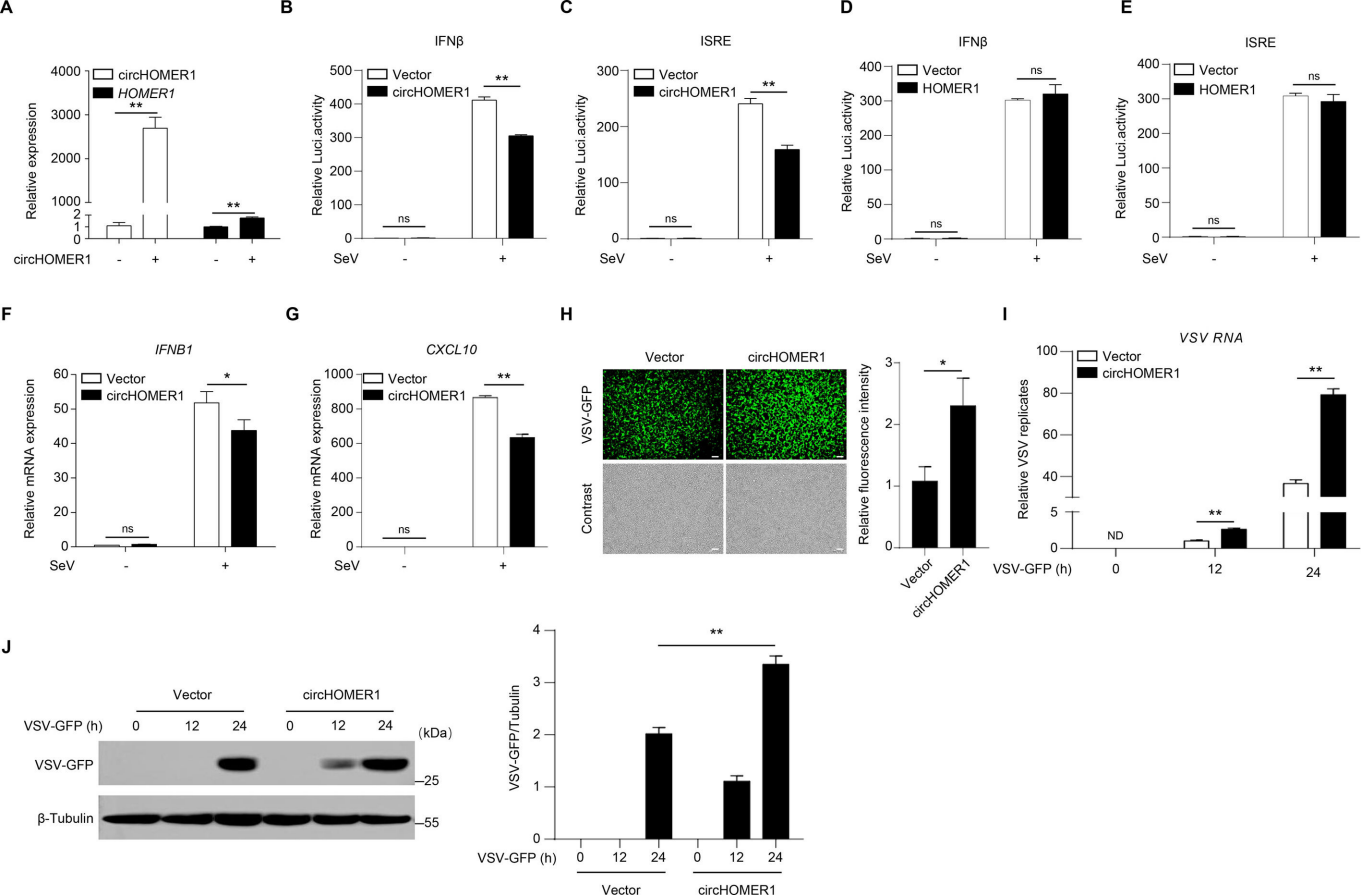
circHOMER1 inhibits virus-induced innate immunity. (**A**) qRT-PCR analyses of the relative expression of circHOMER1 or *HOMER1* in HEK293T cells transfected with the vector or circHOMER1 overexpression plasmid. GAPDH was used as a basal control; (**B and C**) The relative luciferase activity of the IFNβ (**B**) and ISRE (**C**) reporter in HEK293T cells transfected with the vector or circHOMER1 overexpressing plasmid was measured at 24 h after SeV infection. (**D and E**) The relative luciferase activity of the IFNβ (**D**) and ISRE (**E**) reporter in HEK293T cells transfected with the vector or *HOMER1* overexpressing plasmid at 24 h after SeV infection; (**F and G**) qRT-PCR analyses of the relative expression of *IFNB1* (F) and *CXCL10* (G) in HEK293T cells transfected with the vector or circHOMER1 overexpressing plasmid at 12 h after SeV infection. GAPDH was used as a basal control. (**H**) Fluorescence microscopy analyses of the expression level of GFP to monitor the replication of VSV-GFP in HEK293T cells transfected with the vector or circHOMER1 overexpressing plasmid at 24 h after VSV-GFP infection. scale bar, 100 µm. Relative fluorescence intensity were quantified by “ImageJ” software (right). (**I and J**) qRT-PCR (**I**) and WB (**J**) analyses were also used to monitor the replication of VSV-GFP in HEK293T cells. Protein expression levels were quantified by “ImageJ” software (right).

The above data showed that both human and mouse circHOMER1 were upregulated upon viral infection. The role of circHOMER1 in the antiviral innate immune response was then investigated. To this end, we constructed the overexpressing plasmid of circHOMER1, which significantly increased the intracellular expression of circHOMER1 after transfection (Fig. 4A). Dual luciferase reporter assay indicated that overexpression of circHOMER1 inhibited SeV triggered activation of IFN-β reporter and the interferon-stimulated response element (ISRE) reporter (Fig. 4B and C). While, overexpression of the linear transcript *HOMER1* had no effect on virus induced IFN-β and ISRE activation (Fig. 4D and E). Concordantly, circHOMER1 dampened the transcription of *IFNB1* and *CXCL10* after viral infection (Fig. 4F and G). To assess the functional consequences, the viral infection was examined by infection with VSV-GFP, which carries a GFP tag to monitor viral infection. In line with the reporter assay and downstream gene expression, overexpression of circHOMER1 promoted the replication of VSV-GFP as assessed by fluorescence microscopy (Fig. 4H), qRT-PCR (Fig. 4I), and WB (Fig. 4J). Taken together, circHOMER1, but not *HOMER1*, is a negative modulator in antiviral innate immunity.

### Knockdown of circHOMER1 augments virus-induced innate immunity

**Fig 5:**
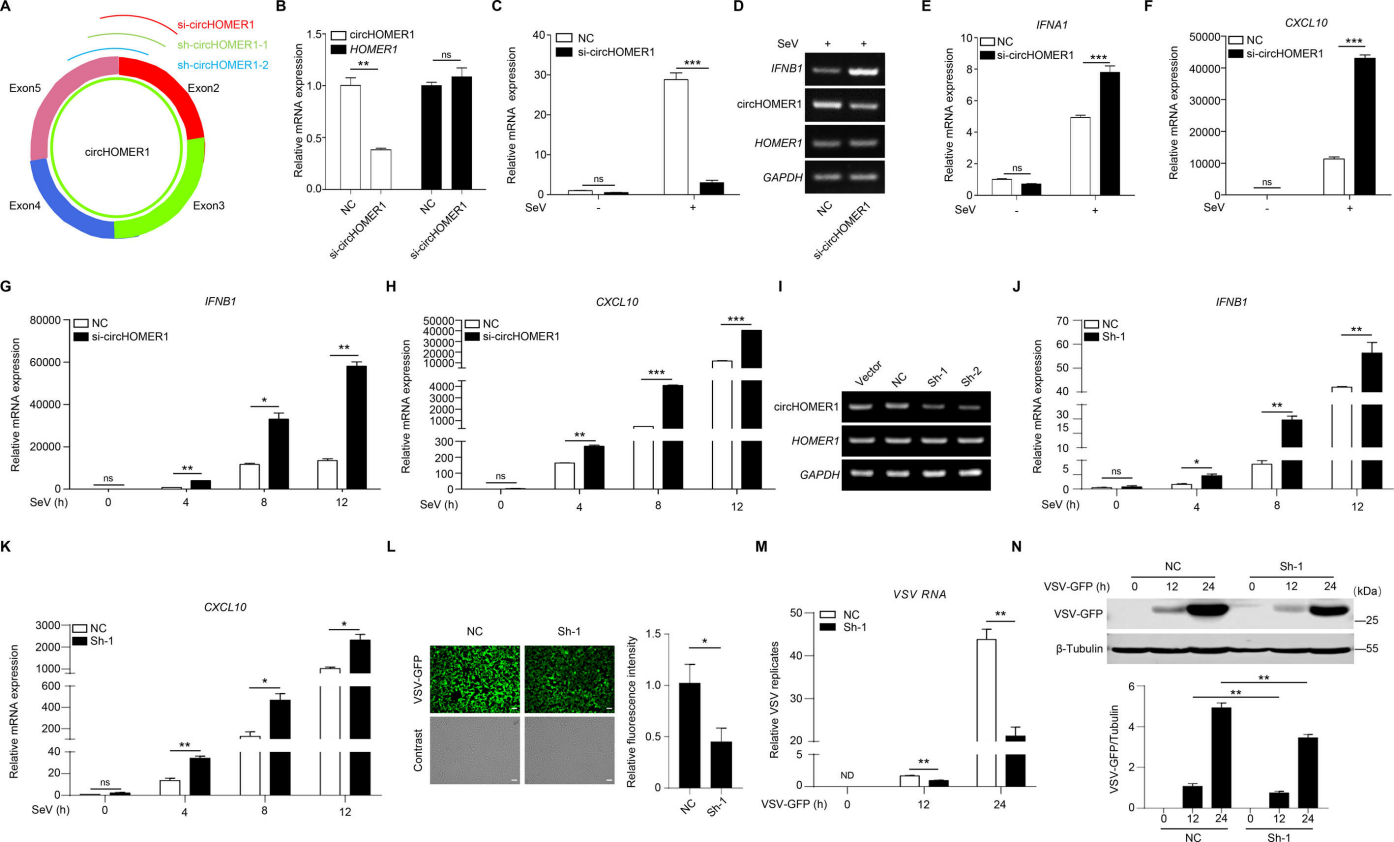
Knockdown of circHOMER1 augments virus-induced innate immunity. (**A**) Schematic diagram exhibiting the targeting sites of circHOMER1-siRNA and circHOMER1-shRNA. (**B**) Relative expression of circHOMER1 and *HOMER1* was analyzed by qRT-PCR in HEK293T cells transfected with NC or circHOMER1-siRNA. (**C**) qRT-PCR analyses of the relative expression of circHOMER1 in HEK293T cells transfected with NC or circHOMER1-siRNA 24 h after SeV infection. (**D**) End-point PCR analyses of the relative expression of *IFNB1*, circHOMER1, and *HOMER1* in HEK293T cells transfected with NC or circHOMER1-siRNA 24 h after SeV infection. (**E and F**) qRT-PCR analyses of the relative expression of *IFNA1* (**E**) and *CXCL10* (**F**) in HEK293T cells transfected with NC or circHOMER1-siRNA at 24 h after SeV infection. (**G and H**) qRT-PCR analyses of the relative expression of *IFNB1* (**G**) and *CXCL10* (**H**) in H460 cells transfected with NC or circHOMER1-siRNA after SeV infection for indicated hours. GAPDH was used as a basal control. (**I**) Relative expression of circHOMER1 and *HOMER1* was analyzed by end-point PCR in HEK293T cells transfected with vector, NC, or circHOMER1-shRNA. (**J and K**) qRT-PCR analyses of the relative expression of *IFNB1* (**J**) and *CXCL10* (**K**) in HEK293T cells transfected with NC or circHOMER1-shRNA after SeV infection for indicated hours. GAPDH was used as a basal control. (**L**) Fluorescence microscopy analyses of the expression level of GFP to monitor the replication of VSV-GFP in HEK293T cells transfected with NC or sh-circHOMER1-1 at 24 h after VSV-GFP infection, scale bar, 100 µm. Relative fluorescence intensity was quantified by “ImageJ” software (right). (**M and N**) qRT-PCR (**M**) and WB (**N**) analyses of the expression level of GFP to monitor the replication of VSV-GFP in HEK293T cells transfected with NC or sh-circHOMER1-1 after VSV-GFP infection for indicated hours. Protein expression levels were quantified by “ImageJ” software(bottom).

To define the physiological functions of endogenous circHOMER1 in the antiviral innate immunity, we designed siRNA oligos and constructed shRNA plasmids (#1, #2) targeting the back-splicing site of circHOMER1 (Fig. 5A). The circHOMER1 siRNA oligos specifically reduced circHOMER1 levels without affecting the linear *HOMER1* (Fig. 5B). The siRNA oligos also significantly attenuated the induced the expression of circHOMER1 upon SeV infection (Fig. 5C) and amplified SeV-induced transcription of *IFNB1*, *IFNA1,* and *CXCL10* in HEK293T cells (Fig. 5D, E and F). Similar effects were recapitulated in H460 cells (Fig. 5G and H). The silencing efficiency of circHOMER1 shRNA #1, #2 was validated (Fig. 5I). Knockdown of circHOMER1 by shRNA enhanced the transcription of *IFNB1* and *CXCL10* (Fig. 5J and K). Consistently, VSV-GFP replication was significantly suppressed by circHOMER1 knockdown as assessed by fluorescence microscopy (Fig. 5L), qRT-PCR (Fig. 5M), and WB (Fig. 5N) in HEK293T cells. Therefore, knockdown of circHOMER1 augments the antiviral innate immunity.

### circHOMER1 modulates the antiviral immune response by miR-145-3p/OTUD7B/IRF3 signaling axis

**Fig 6:**
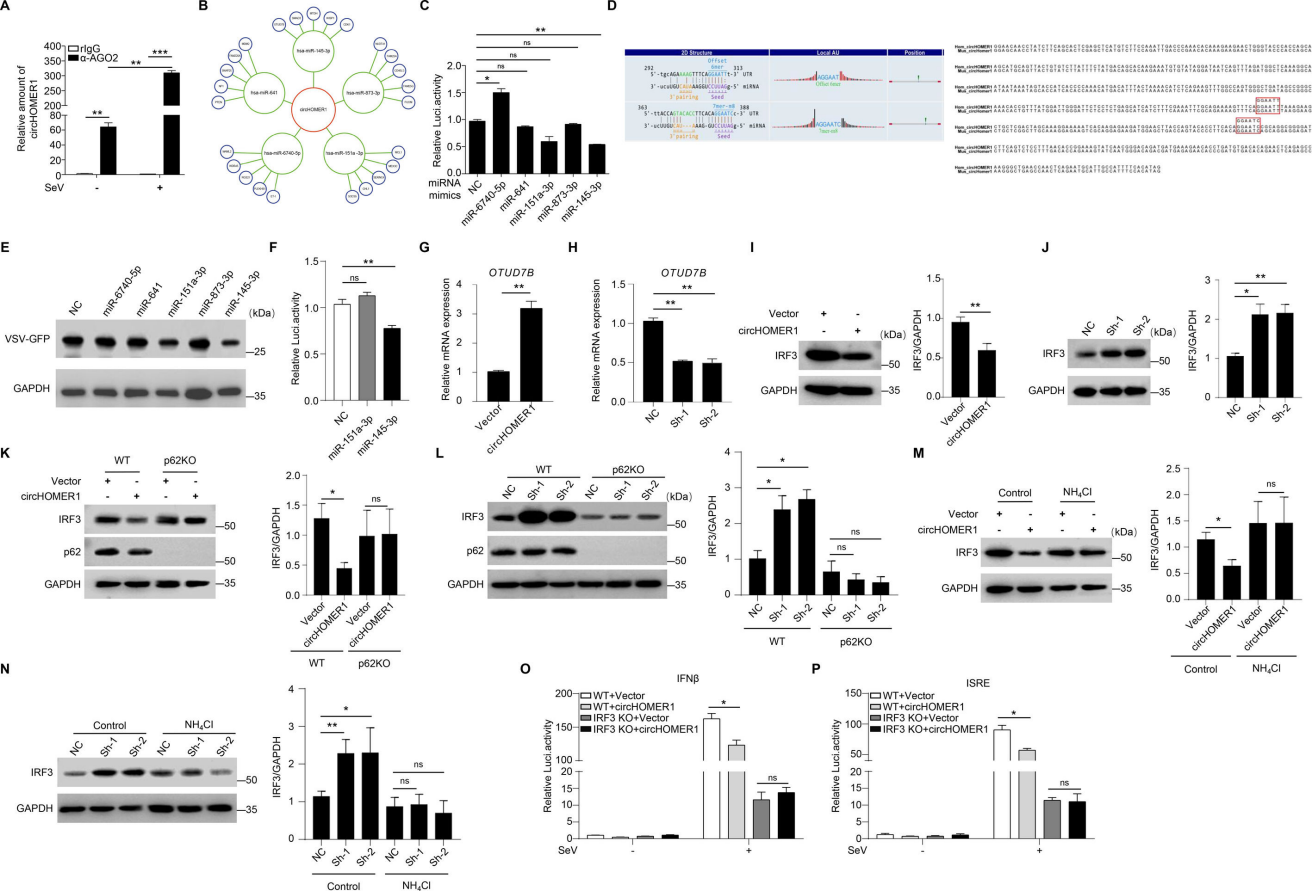
circHOMER1 modulates the antiviral immune response by miR-145-3p/OTUD7B/IRF3 signaling axis. (**A**) RIP analysis of the interaction between circHOMER1 and AGO2 in HEK293T cells. (**B**) The constructed ceRNA network of circRNA-miRNA-mRNA interactions. (**C**) The relative luciferase activity of the circHOMER1 reporter in HEK293T cells transfected with the indicated miRNA mimics. Firefly luciferase activity was normalized to Renilla luciferase activity. (**D**) The specific binding sites of miR-145-3p and circHOMER1 predicted by TargetScan and miRanda database and sequence identity analysis of potential binding sites of human and mouse circHOMER1. Potential binding sites were marked with a red box. (**E**) WB analyses of GFP expression to monitor the replication of VSV-GFP in HEK293T cells transfected with the indicated miRNA mimics at 24 h after VSV-GFP infection. (**F**) The relative luciferase activity of the OTUD7B 3’UTR reporter in HEK293T cells transfected with the indicated miRNA mimics. (**G and H**) qRT-PCR analyses of the relative expression of OTUD7B in HEK293T cells transfected with vector or circHOMER1 (G), NC, or circHOMER1-shRNA (H). (**I and J**) WB analyses of IRF3 expression in HEK293T cells transfected with vector or circHOMER1 (I), NC, or circHOMER1-shRNA (J). Protein expression levels were quantified by “ImageJ” software (right). (**K and L**) WB analyses of the IRF3 expression in WT or SQSTM1/p62KO HeLa cells transfected with vector or circHOMER1 (K), NC, or circHOMER1-shRNA (L). Protein expression levels were quantified by “ImageJ” software(right). (**M and N**) WB analyses of the relative expression of IRF3 in HEK293T cells, which transfected with vector or circHOMER1 (M), NC, or circHOMER1-shRNA (N) and subsequently treated with control or NH_4_Cl (10 mM). Protein expression levels were quantified by “ImageJ” software(right). (**O and P**). The relative luciferase activity of the IFNβ (**O**) and ISRE (**P**) reporter in WT or IRF3 KO HEK293T cells transfected with vector or circHOMER1 overexpressing plasmid at 24 h after SeV infection.

The above results showed that circHOMER1 was primarily localized in the cytoplasm. It has been proposed that most cytoplasmic circRNAs can function as ceRNAs through Argonaute 2 (AGO2) (26, 27). RNA immunoprecipitation (RIP) assays were performed and revealed that circHOMER1 can interact with AGO2, and this interaction was enhanced by SeV infection (Fig. 6A). This further implied that circHOMER1 may act as a ceRNA in the antiviral immune response. According to bioinformatics analysis, circHOMER1 may interact with a number of miRNAs (Fig. 6B), among which miR-145-3p was found to bind and to have the most inhibitory effect on the activity of the circHOMER1 reporter (Fig. 6C). The specific binding sites were predicted to be the 292–313 nt and/or 363–388 nt regions of circHOMER1 (Fig. 6D). And the specific binding sites were conserved in human and mouse circHOMER1 (Fig. 6D).

The addition of miR-145-3p mimics strongly inhibited the replication of VSV-GFP (Fig. 6E). According to TargetScan analysis, one of the possible targets of miR-145-3p is the deubiquitinase OTUD7B, which is known to inhibit type I IFNs signaling by deubiquitinating SQSTM1/p62 and promoting autophagy-dependent degradation of IRF3 (28). We then constructed the pGL3-basic-3′UTR-OTUD7B luciferase reporter plasmid to test whether the miRNA can target it. The luciferase reporter assay revealed that miR-145-3p can inhibit the luciferase activity, whereas, hsa-miR-151a-3p had no effect (Fig. 6F), indicating that miR-145-3p may bind to OTUD7B 3′UTR. To demonstrate whether circHOMER1 was the sponge for miR-145-3p to control the expression of OTUD7B mRNA, OTUD7B mRNA levels were examined when circHOMER1 was overexpressed or knocked down. As expected, the overexpression of circHOMER1, indeed, increased the mRNA expression of *OTUD7B* (Fig. 6G). In contrast, knockdown of circHOMER1 decreased *OTUD7B* expression (Fig. 6H).

OTUD7B is known to regulate the protein level of IRF3 in an autophagy-dependent manner (28). We, therefore, sought to determine whether IRF3 protein levels were altered by circHOMER1. Compared to the control group, overexpression of circHOMER1 resulted in decreased IRF3 protein levels (Fig. 6I). On the other hand, circHOMER1 knockdown increased IRF3 protein levels (Fig. 6J). As mentioned above, OTUD7B promoted IRF3 degradation in a SQSTM1/p62-dependent manner(28). SQSTM1/p62 KO cells were used to investigate the regulatory functions of cricHOMER1 on the protein level of IRF3. In WT cells, circHOMER1 promoted the reduced expression of IRF3. This phenomenon was not observed in SQSTM1/p62 KO cells (Fig. 6K and L). Ammonium chloride (NH_4_Cl) is a known inhibitor of autophagy/lysosome (14, 29). We then sought to explore whether the function of circHOMER1 was dependent on autophagy-induced degradation. After treatment with NH_4_Cl, circHOMER1 cannot promote the reduced expression of IRF3 (Fig. 6M and N). Moreover, IRF3 KO cells were also used to examine the inhibitory function of cricHOMER1 in innate immunity. It was shown that the inhibitory effects of circHOMER1 were not observed in IRF3 KO cells (Fig. 6O and P), confirming that IRF3 was the target for circHOMER1. Thus, circHOMER1 modulated the antiviral immune response through miR-145-3p/OTUD7B/IRF3 signaling axis.

### circHOMER1 impairs RIG-I sensing by disrupting G3BP1-RIG-I complex formation

**Fig 7:**
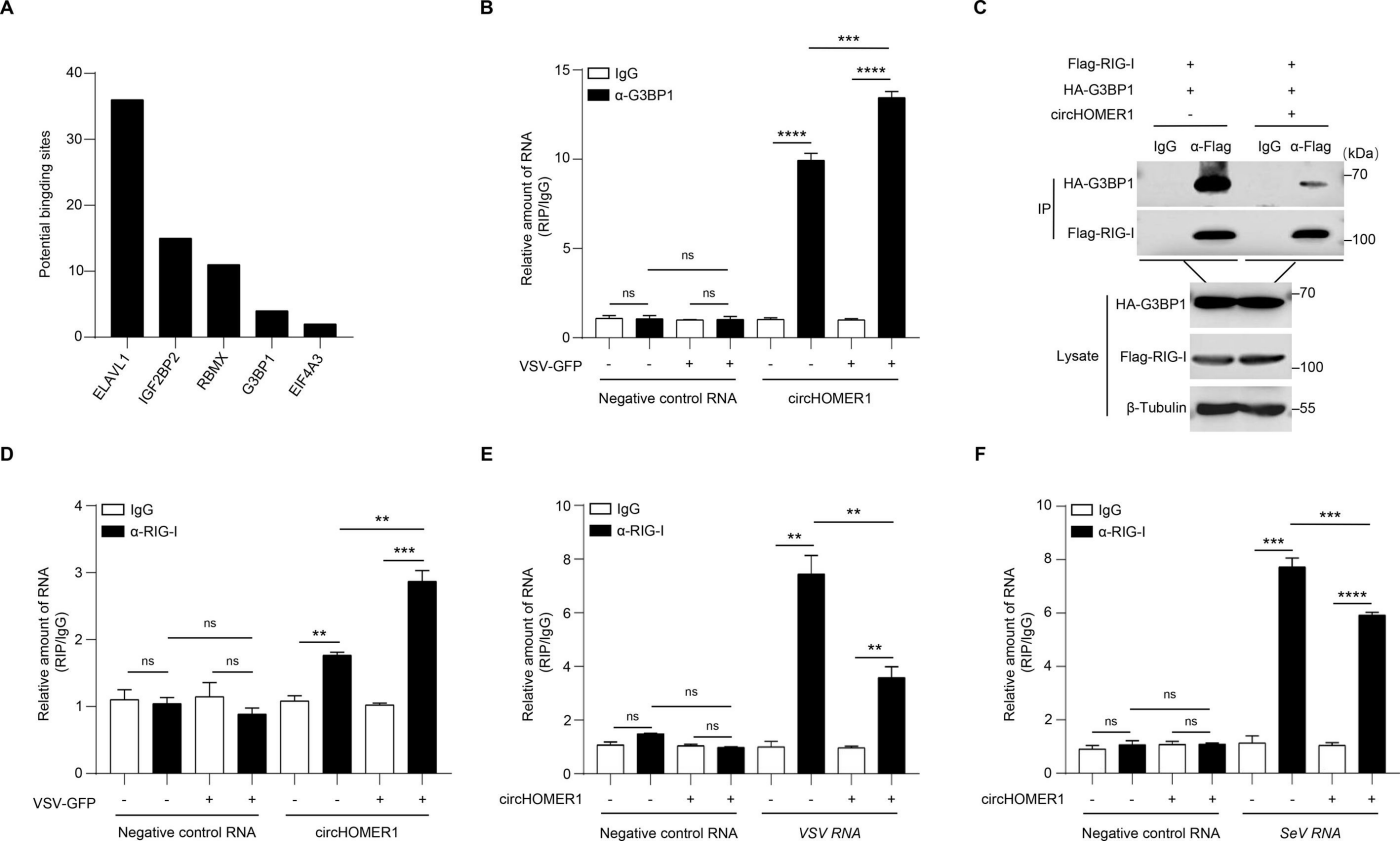
circHOMER1 impairs RIG-I sensing by disrupting G3BP1-RIG-I complex formation. (A) The potential binding sites of circHOMER1 and RBPs predicted by the ENCORI database. (B) RIP analyses of the interaction between circHOMER1 and G3BP1 in HEK293T cells infected with or without VSV-GFP. Negative control RNA was GAPDH. (C) IP analyses of the interaction between G3BP1 and RIG-I in HEK293T cells transfected with vector or circHOMER1 overexpressing plasmid. (D) RIP analyses of the interaction between circHOMER1 and RIG-I in HEK293T cells infected with or without VSV-GFP. Negative control RNA was GAPDH. (E) RIP analysis of the interaction between VSV RNA and RIG-I in HEK293T cells transfected with vector or circHOMER1 overexpressing plasmid after infected with VSV-GFP for 6 h. Negative control RNA was GAPDH. (F) RIP analysis of the interaction between SeV RNA and RIG-I in HEK293T cells transfected with vector or circHOMER1 overexpressing plasmid after infected with VSV-GFP for 6 h. Negative control RNA was GAPDH.

Certain circRNAs are capable of attaching RNA-binding proteins and control RBP functions (1). Computational prediction identified G3BP1, a stress granule scaffold protein critical for RIG-I-dependent immune activation (30, 31), as one of the candidate circHOMER1-associated proteins (Fig. 7A). To validate this association, the interaction of circHOMER1 and G3BP1 was detected with or without viral infection. It was shown that circHOMER1 can, indeed, bind to G3BP1 and that this association was enhanced after VSV-GFP infection (Fig. 7B). Strikingly, circHOMER1 expression disrupted G3BP1-RIG-I complex formation (Fig. 7C), which is a prerequisite for efficient viral RNA sensing (30). In addition to being a reader of foreign dsRNA nucleic acids, RIG-I is also known as a general reader of circRNAs (32). It was found that RIG-I can bind circHOMER1, and this binding was enhanced by VSV-GFP infection (Fig. 7D). Notably, the binding of RIG-I to foreign viral RNA (VSV-GFP and SeV) was also reduced in the presence of circHOMER1 by endogenous RNA immunoprecipitation (RIP) (Fig. 7E and F). Thus, circHOMER1 can sequester the G3BP1-RIG-I complex, limits the accessibility of RIG-I to viral ligands, and thereby attenuating viral RNA-induced RLR-dependent signaling.

### circHomer1 exacerbates viral pathogenesis by suppressing innate immunity *in vivo*

**Fig 8:**
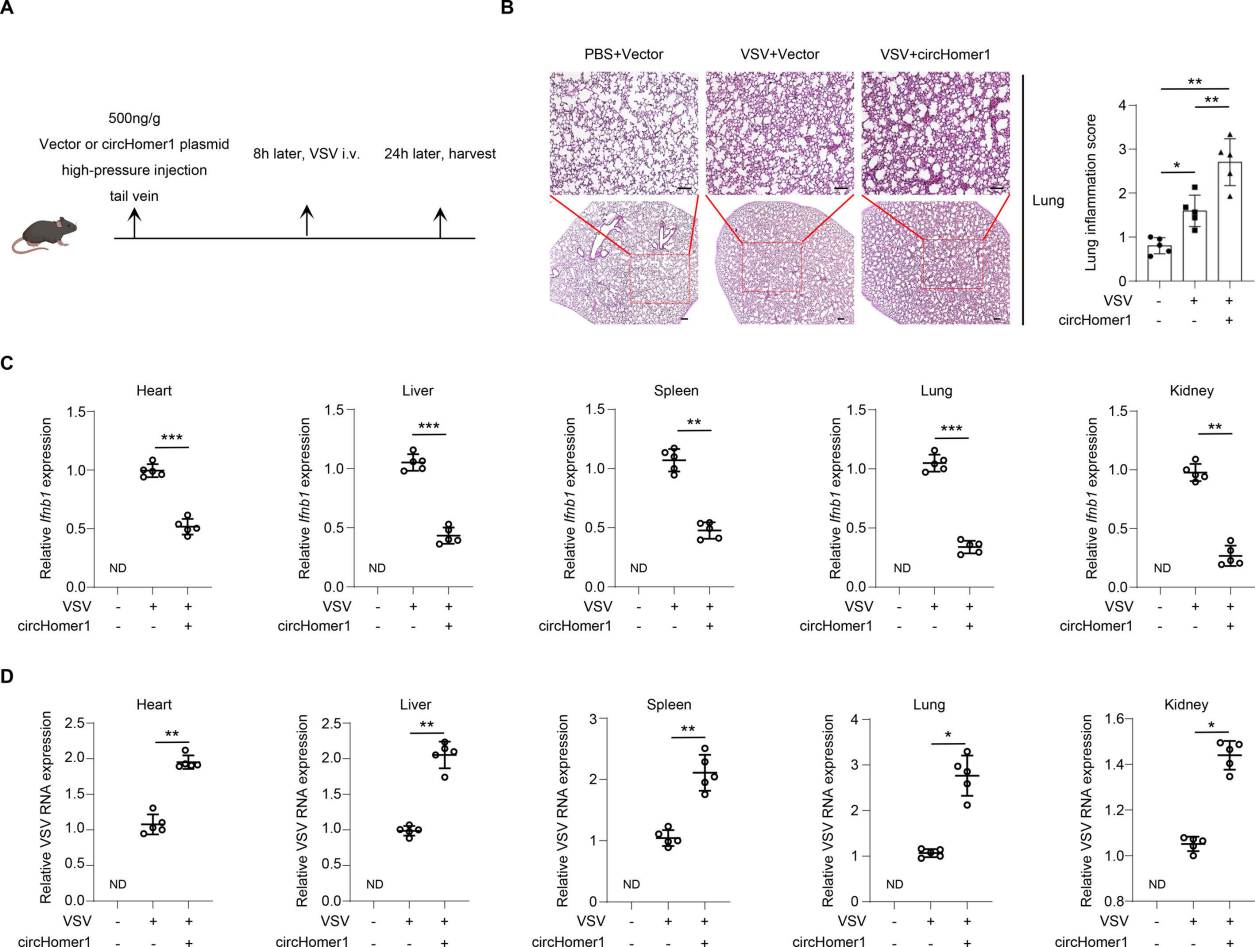
circHomer1 exacerbates viral pathogenesis by suppressing innate immunity *in vivo*. (**A**) Schematic diagram of the treatment strategy for the *in vivo* study. (**B**) Representative HE staining of the lung tissue from vector or circHomer1-overexpressing mice infected with VSV (2 × 10^7^ pfu/g); scale bar, 100 µm. Inflammation scores of lung tissue sections shown in right (*n* = 5 mice per group). (**C**) Relative expression of *Ifnb1* was analyzed by qRT-PCR in heart, liver, spleen, lung, and kidney of mice for the indicated treatment (*n* = 5 mice per group). (**D**) Relative expression of *VSV RNA* was analyzed by qRT-PCR in heart, liver, spleen, lung, and kidney of mice for indicated treatment (*n* = 5 mice per group).

The above results showed that circHOMER1 inhibited virus-induced innate immunity in human cell lines. To validate the immunosuppressive role of circHOMER1 *in vivo*, a mice VSV infection model was employed. Hydrodynamic delivery of a circHomer1-overexpressing plasmid resulted in exacerbated lung pathology, characterized by heightened inflammatory infiltration and tissue damage (Fig. 8A and B). qRT-PCR revealed that circHomer1 overexpressing mice showed reduced VSV-induced transcription of *Ifnb1* mRNA in heart, liver, spleen, lung, and kidney (Fig. 8C), concomitant with elevated VSV RNA loads in these organs (Fig. 8D), suggesting that circHomer1 inhibited the antiviral immune response and promoted viral replication *in vivo*. This reinforced the notion that circHOMER1 was a negative modulator in antiviral innate immune immunity.

## DISCUSSION

Emerging evidence suggests that circRNAs are involved in various physiological or pathological processes (33), with a particular emphasis on their function in the innate immune response (4, 6). We performed circRNA microarray screening to characterize unidentified circRNAs in antiviral innate immunity. Among the differentially expressed circRNAs, circHOMER1 was significantly upregulated upon SeV infection. Then using different stimuli, circHOMER1 can be upregulated by SeV, VSV, HSV-1, IFN-α, and IFN-β in different cell types. circHOMER1 was conserved across species. Notably, circHomer1 was also upregulated by viral infection. Although a recent study reported that stimulation of HeLa cells with RNA viruses or RNA virus mimics may lead to the degradation and reduction of global circRNAs (6). The half-life of endogenous circHOMER1 was not shortened compared to the uninfected group. Viral infection did not lead to the degradation of circHOMER1. Instead, circHOMER1 expression was enhanced by viral infection or type I IFNs stimulation. In recent years, several virus-induced circRNAs have been reported and discovered to be involved in the positive or negative regulation of innate immunity. For instance, circCBL, upregulated by *Sininiperca chuatsi rhabdovirus* (SCRV) and LPS stimulation, favorably controls the antiviral innate immunity by absorbing miR-125a-1-3p to enhance the expression of stimulator of interferon response cGAMP interactor 1 (STING) (34). circMORC3, also induced by SCRV, encodes a peptide of 84 amino acids that interacts with TIR domain-containing adaptor inducing interferon-β (TRIF), facilitates its autophagic degradation, and inhibits antiviral innate immunity (35). circMerTK, which is induced by influenza A virus (IAV), suppresses antiviral innate immunity (36). Given the large number of differentially expressed host circRNAs, the known functions of circRNAs in regulating innate immunity are very limited. In addition, the back-splice sequence of circHOMER1 was conserved in humans and mice by sequence alignment and Sanger sequencing. And the full-length sequences of circHOMER1 from human and mouse shared an identity of 93%. As a novel virus and type I IFNs-induced circRNA, the functions of circHOMER1 have been reported in relation to the nervous system and neurological diseases, and its immune functions are still unknown.

Using an overexpression or knockdown experimental system, circHOMER1 was shown to have negative regulatory functions in the antiviral immune response *in vitro* or *in vivo*. It suppressed IFN-β and ISRE reporter activation and promoted viral replication. The next question is how circHOMER1 exerts its inhibitory role in innate immunity. Nuclear-cytoplasmic fractionation revealed that circHOMER1 was mainly predominantly cytoplasmic localization l. The coding potential of circHOMER1 was also analyzed. It does not contain an IRES and has no coding potential. An important mode of action for cytoplasmic circRNA is ceRNA (27, 37). AGO2 immunoprecipitation experiments showed that SeV infection enhanced this association between AGO2 and circHOMER1, suggesting that circHOMER1 may act as a ceRNA for miRNAs. By bioinformatic analysis was used to construct the circHOMER1 ceRNA interaction network. Further dual luciferase reporter assays validated that circHOMER1 acted as a sponge for miR-145-3p, which was involved in the regulation of viral replication or antiviral innate immunity.

Only a limited number of genes have been identified as targets for miR-145-3p. The primary documented roles of miR-145-3p are acting as a tumor suppressor in cancer or a diagnostic marker in certain disorders (38–40). In this study, miR-145-3p was discovered to inhibit viral replication and boost antiviral innate immunity. This is the first report on the function of miR-145-3p in innate immunity. Since there are so few known targets of miR-145-3p, we attempted to identify novel targets for miR-145-3p in innate immunity. Through bioinformatic analysis, OTUD7B was one of the potential targets, which has been reported to negatively regulate antiviral innate immunity by deubiquitinating SQSTM1/p62 and facilitating IRF3 degradation (28). The binding of miR-145-3p to OTUD7B was then demonstrated using the OUTD7B 3′UTR luciferase plasmid, and circHOMER1 can promote the degradation of SQSTM1/p62 and IRF3. In SQSTM1/p62 KO cells, IRF3 protein levels were not altered in the presence or absence of circHOMER1 or knockdown of circHOMER1 expression, suggesting that circHOMER1 may negatively regulate the antiviral innate immunity through the miR-145-3p/OTUD7B/IRF3 axis.

Previously, miR-145-3p was reported to promote autophagy (41). Our findings revealed an additional mechanism by which miR-145-3p promoted autophagy, namely, the miR-145-3p/OTUD7B/p62 axis. We can now exclude the possibility that circHOMER1 or miR-145-3p has any additional unidentified targets in antiviral innate immunity, as circRNAs and miRNAs may have numerous targets. Furthermore, miRNAs including miR-217, miR-23b, and miR-138-5p have been reported to be direct targets of circHOMER1. miR-217 has been shown to target TBK1, a kinase critical for innate immunity (42). miR-23b has the ability to modulate antiviral innate immunity by downregulating cGAS expression (43). It will be important in future studies to confirm whether circHOMER1 acts on these miRNAs to control downstream gene expression and modify antiviral innate immunity.

RIG-I-like receptors (RIG-I and MDA5) are PRRs that are responsible for sensing foreign dsRNAs and activating innate immunity (44–46). In addition, RIG-I is considered to be the general reader of circRNAs (32). The m^6^A modification enables RIG-I to distinguish between self and foreign circRNAs. Only foreign circRNAs without m^6^A modification can bind and activate RIG-I (47). The present study revealed that circHOMER1 can also interact with RIG-I and that this interaction was enhanced by viral infection. And the binding of circHOMER1 to RIG-I inhibited the recognition of RIG-I to foreign viral RNAs. In predicting the potential RBPs of circHOMER1, G3BP1 was a candidate. It is well known that G3BP1 binds to RIG-I and stimulates RIG-I-dependent signaling (30). circHOMER1 was observed to bind to G3BP1, and this interaction was also enhanced by viral infection. In the presence of circHOMER1, the association between G3BP1 and RIG-I was reduced.

EIF4A3 is another RBP of circHOMER1. It is known that eIF4A3 is important for the biogenesis of circHOMER1 in the nervous system (13). We next sought to determine whether eIF4A3 was also critical for the biogenesis of circHOMER1 upon viral infection. Upon SeV infection, the binding of circHOMER1 and eIF4A3 was significantly enhanced. Knockdown of eIF4A3 dramatically reduced the biogenesis of circHOMER1 induced by SeV infection. This suggested that eIF4A3 was also essential for the biogenesis of circHOMER1 after viral infection. EIF4A3 has been reported to negatively regulate antiviral innate immunity by suppressing IRF3 activation (48). According to our findings, it is possible that eIF4A3 promotes the expression level of circHOMER1 and acts as a negative modulator through circHOMER1-mediated inhibitory functions in innate immunity. RBPs such as ELAV like RNA-binding protein 1 (ELAVL1) and insulin like growth factor 2 mRNA-binding protein 2 (IGF2BP2) may also interact with circHOMER1 and mediate the suppressive function of circHOMER1. This needs to be further investigated in the future.

The biogenesis process of circHOMER1 and its linear transcript counterpart after viral stimulation was also examined. After SeV infection, the biosynthesis of circHOMER1 was increased, whereas both the linear transcript *HOMER1* and the pre-mRNA were decreased. The function of the linear part of *HOMER1* in the regulation of innate immunity was also investigated. It was shown that only circHOMER1, but not *HOMER1*, is involved in the regulation of innate immunity.

Previously, it has been reported that circHOMER1 can be upregulated by methamphetamine (METH), which has broad immunosuppressive functions (20, 21). However, the molecular mechanism of METH-induced immune suppression remains unclear. Based on our findings, it is possible that METH suppresses innate immunity by upregulating circHOMER1(20). In our established viral encephalitis model, circHomer1 was upregulated by RNA or DNA viruses. As a neuronally enriched suppressive circRNA in innate immunity and inflammation, circHOMER1 may be important in the prevention of viral encephalitis. Dysregulation of circHOMER1 may be associated with the development of viral encephalitis and related diseases.

In conclusion, circHOMER1 was a novel interferon-stimulated circRNA that can negatively regulate the antiviral innate immunity through the miR-145-3p/OTUD7B/IRF3 axis. It also interfered with the interaction between G3BP1 and RIG-I and inhibited the recognition of foreign RNAs by RIG-I. Therefore, circHOMER1 was an important modulator in immune homeostasis. Dysfunction of circHOMER1 may be associated with aberrant innate immune responses and excessive inflammation-related diseases.

## MATERIALS AND METHODS

### Cell lines and culture

HEK293T, HEK293 cells, HEK293T IRF3 KO cells, iBMDMs, HeLa cells, African green monkey kidney cell line Vero cells, and HeLa SQSTM1/p62 KO cells were cultured in Dulbecco modified Eagle medium (DMEM; Thermo Fisher Scientific), which contained 10% heat-inactivated fetal bovine serum (FBS; CellMax), 100 U/mL penicillin, and 100 ng/mL streptomycin (Thermo-Fisher Scientific). H460, A549, and H1299 were maintained in RPMI-1640 medium (Macgene) supplemented with 10% FBS and penicillin-streptomycin. The 0.25% rypsin-EDTA solution (Macgene) was employed to detach adherent cells from the flasks or dishes. All these cell lines were cultured under standard culture conditions (5% CO_2_, 37°C).

### Viruses

The SeV was propagated by inoculation into the allantoic cavities of embryonated chicken eggs. HSV-1, HSV-1-GFP, VSV, and VSV-GFP were amplified in Vero cells. The viral titers of SeV, HSV-1, HSV-1-GFP, VSV, and VSV-GFP were determined by the tissue culture infectious dose 50% (TCID50) on L929 cells as described (49–51). The cells were infected with SeV, HSV-1, HSV-1-GFP, VSV, or VSV-GFP for the indicated time points.

### Reagents and antibodies

ActD (31852-29-6) and RNase R (86828-69-5) were obtained from Merck and Epicentre, respectively. Poly(I:C) (tlrl-picw) and poly(dA:dT) (tlrl-patn) were from InvivoGen. Recombinant human IFN-α2 (592702) was from BioLegend, and IFN-β (300-02B.C.BC-20) was from PeproTech. Opti-MEM (31985070) was purchased from Thermo-Fisher Scientific. Anti-GFP (66002-1-Ig), anti-GAPDH (60004-1-AP), and anti-AGO2 (10686-1-Ig) were from Proteintech. Anti-Flag (M185-3L) was from MBL. Anti-HA (M20003S) was from Abmart. The anti-RIG-I (D14G6) and anti-IRF3 (D83B9) antibodies were obtained from Cell Signaling Technology. And the HRP-conjugated anti-mouse IgG (BF03001) and anti-rabbit IgG (D83B9) were from Biodragen Immunotechnologies Co., Ltd.

### Plasmids, RNA oligos, and miRNA mimics

The full-length circHOMER1 sequence was inserted into the pcDNA3.1 vector with flanking inverted repeats that facilitate intracellular circularization. And the full-length sequence of circHOMER1 was subcloned into the 3′-UTR of the pGL3-Basic vector (Promega) to construct pGL3-Basic-3′UTR-circHOMER1. The pcDNA3.1-Flag-G3BP1 were provided by Dr. Jianyuan Luo. The 3′UTR sequence of OTUD7B was inserted into the 3′UTR of the pGL3-Basic vector (Promega) to construct pGL3-Basic-3′UTR-OTUD7B. The shRNA sequences were annealed and subsequently inserted into pLKO.1 vector. All these constructs were verified by sequencing. The siRNA/shRNA oligos and miRNA mimics were synthesized by GenePharma. The sequences were presented as follows: circHOMER1-NC, 5′-UUCUCCGAACGUGUCACGU-3′; circHOMER1-siRNA-#1, 5′-CCAUUUUCACAUAGGGAACTT-3′; circHOMER1-shRNA-#1, 5′-ACATAGGGAACAACCTATCTT-3′; circHOMER1-shRNA-#2, 5′-TTCACATAGGGAACAACCTAT-3′; hsa-miR-6740-5p mimics, 5′-AGUUUGGGAUGGAGAGAGGAGA-3′; hsa-miR-641 mimics, 5′-AAAGACAUAGGAUAGAGUCACCUC-3′; hsa-miR-151a-3p mimics, 5′-CUAGACUGAAGCUCCUUGAGG-3′; hsa-miR-873-3p mimics, 5′-GGAGACUGAUGAGUUCCCGGGA-3′; and hsa-miR-145-3p mimics, 5′-GGAUUCCUGGAAAUACUGUUCU-3′;

### RNA preparation and quantitative real-time PCR

RNA preparation and qRT-PCR were performed as previously described (49). Gene expression was quantified as the level of the indicated genes relative to that of *GAPDH* or β*-Actin* and calculated through the 2^-ΔΔCt^ method. The primers used are listed as follows: circHOMER1-F-Divergent, GAACTTACCAGTACACCTTCACAGG; circHOMER1-R-Divergent, GGGTACCCAGTTCTTCTTTGTGT; circHOMER1-F-Convergent, CACTCGAGCTCATGTCTTCC; circHOMER1-R-Convergent, AGACACAGTAACTGCATGCTT; HOMER1-F, GGTGGAAAAGCAACTGCGAA; HOMER1-R, ACATGAGCTCGAGTGCTGAA; Pre-HOMER1-F, AGCAAGCATGCAGTTACTGTG; Pre-HOMER1-R, AAGCAAAACCAGCCAAATCATTC; circHomer1-F-Divergent, CATTTCCACATAGGGAGCAACC; circHomer1-R-Divergent, ATCAGCCCATTGGCCAAACT; Homer1-F, GTGTCAGCGCGAGTGAAATC; Homer1-R, ACATGAGCTCGAGTGCTGAA; VSV-RNA-F, ACGGCGTACTTCCAGATGG; VSV-RNA-R, CTCGGTTCAAGATCCAGGT; CXCL10-F, GTGGCATTCAAGGAGTACCTC; CXCL10-R, TGATGGCCTTCGATTCTGGATT; IFNB1-F, ACTGCCTCAAGGACAGGATG; IFNB1-R, GGCCTTCAGGTAATGCAGAA; IFNA1-F, GCCTCGCCCTTTGCTTTACT; IFNA1-R, CTGTGGGTCTCAGGGAGATCA; OTUD7B-F, GTCAGATTTTGTCCGTTCCACA; OTUD7B-R, CATGGACTTGACGTAGCTGTT; Mouse-Ifnb1-F, CAGCTCCAAGAAAGGACGAAC; Mouse-Ifnb1-R, GGCAGTGTAACTCTTCTGCAT; GAPDH-F, ACCCACTCCTCCACCTTTGA; GAPDH-R, CTGTTGCTGTAGCCAAATTCGT; U6-F, CTCGCTTCGGCAGCACA; U6-R, AACGCTTCACGAATTTGCGT; Mouse-β-Actin-F, AGAGGGAAATCGTGCGTGAC;and Mouse-β-Actin-R, CAATAGTGATGACCTGGCCGT;

### Subcellular fractionation and RNA analysis

Nuclear and cytosolic RNAs were extracted with the PARIS Kit (Thermo Fisher Scientific, AM1921) according to the manufacturer’s instructions. Subsequently, 3 µg nuclear or cytosolic RNAs were reverse-transcribed into cDNA for qRT-PCR quantification respectively as the protocol mentioned above.

### Actinomycin D assay and RNase R treatment

ActD treatment and RNase R digestion were utilized to evaluate the stability of circHOMER1. In the ActD assay, HEK293T cells were treated with ActD (3 µg/mL) for 0, 12, 18, and 24 h. The expression of circHOMER1, *HOMER1,* and *GAPDH* was examined by qRT-PCR or end-point PCR. For the RNase R digestion assay, a portion of the RNA extracted from HEK293T cells was subjected to 3 U/µg RNase R for 0, 10, 20, or 30 min at 37°C.

### Dual-luciferase reporter assays

Dual-luciferase reporter assays were performed as previously described (49). If miRNA mimics bind to circHOMER1 sequences, the level of fluorescence produced by the cells was reduced. The luciferase activity was normalized to *Renilla* luciferase signal.

### RNA immunoprecipitation

All steps were performed under RNase-free conditions. HEK293 cells were harvested and lysed in RIP lysis buffer for 30 min on ice. Twenty percent of the sample was reserved for Western blotting or qRT-PCR analysis, respectively, while the remaining lysates were incubated with protein G-Sepharose beads adsorbing the indicated antibody overnight at 4°C. The beads were then washed three times in RIP lysis buffer and eluted at 100°C for 10 min. The samples were then separated by SDS-PAGE and transferred to nitrocellulose membranes (Cytiva). After blocking with 5% non-fat milk, the membranes were sequentially incubated with antibodies before visualization in ImageQuant LAS 500 (GE Healthcare Life Science).

### Isolation of primary mouse peritoneal macrophages

Wild-type C57BL/6J mice (aged 6–8 weeks) were obtained from Beijing Vital River Laboratory Animal Technology. PMs were harvested through optimized thioglycolate-elicited protocol as previously described (49).

### *In vivo* VSV infection mouse model

The age and sex matched 6- to 8-week-old C57BL/6J mice were divided into two groups and injected with either control or circHomer1-expressing plasmid (500 ng/g/each) via the tail vein. Eight hours later, each mouse was intraperitoneally injected with VSV (1 × 10^7^ pfu/g) into for infection. Twenty-four hours after infection, the mice were euthanized and the organs were harvested. The tissues were then fully ground using a tissue grinder (TIANGEN). The following steps were mentioned in “RNA preparation and quantitative real-time PCR.” Hematoxylin and eosin (H&E) staining was conducted to examine the morphology of the lung tissue sections. The mice were fed under specific-pathogen-free conditions.

### Bioinformatics analyses

The miRNAs of circHOMER1 were predicted by the bioinformatics databases miRanda (52) (https://regendbase.org/tools/miranda) and TargetScan (53) (http://www.targetscan.org/vert_70). The circHOMER1 sequences of human, mouse, and rabbit were downloaded from CircFunBase (54) (https://bis.zju.edu.cn/CircFunBase/). The circHOMER1 sequences of human, mouse, and rabbit were aligned using the ClustalW 2.1 software (http://www.clustal.org/clustal2/). The RNA-binding proteins of circHOMER1 were predicted through the CircInteractome (55) (https://circinteractome.nia.nih.gov) and CSCD (http://gb.whu.edu.cn/CSCD) databases.

### Statistical analyses

All statistical analyses were carried out using GraphPad Prism 8.0 software to evaluate the differences between experimental and control groups. Relative fluorescence intensity and relative protein level were quantified by “ImageJ” software. Data were analyzed by Student’s *t*-test for comparison of two samples. The criterion of statistical significance was considered at *P* < 0.05, while *P* > 0.05 indicates not significant. Data are displayed as the mean ± SD.

## Data Availability

The circRNA microarray data used in this study has been deposited in the NCBI GEO database (GSE296643).
